# The complete chloroplast genome of an endangered species *Cymbidium mastersii* (Orchidaceae)

**DOI:** 10.1080/23802359.2019.1666047

**Published:** 2019-09-19

**Authors:** Fang Zheng, Yun-Yun Pan, Ting-Zhang Li, Gui-Zhen Chen, Xin-Yi Wu, Li-Qiang Li

**Affiliations:** a Key Laboratory of National Forestry and Grassland Administration for Orchid Conservation and Utilization, The National Orchid Conservation Centre of China and The Orchid Conservation and Research Centre of Shenzhen, Shenzhen, China;; b Shenzhen Key Laboratory for Orchid Conservation and Utilization, The National Orchid Conservation Centre of China and The Orchid Conservation and Research Centre of Shenzhen, Shenzhen, China

**Keywords:** *Cymbidium mastersii*, chloroplast genome, phylogenetic

## Abstract

*Cymbidium mastersii* Griff. & Lindl. is an endangered orchid. In this study, we report the complete chloroplast (cp) genome sequence and the cp genome features of *C. mastersii.* The cp genome sequence of *C. mastersii* was 155,362 bp in length. It included one large single-copy region (LSC, 84,465 bp), one small single-copy region (SSC, 20,647 bp), and two inverted repeat regions (IRs, 25,125 bp). The cp genome encoded 130 genes, of which 107 were unique genes (80 protein-coding genes, 23 tRNAs, and 4 rRNAs). The maximum-likelihood phylogenetic analysis showed that *C. mastersii* was a sister of *C. erythraeum* and *C. nanulum*.

*Cymbidium* Swartz, 1799 (Swartz 1799, p. 6), an important genus in Orchidaceae, is native of tropical and subtropical Asia covering North-India, China, Japan, Malaysia, the Philippines, and Borneo islands, and North-Australia (Chen et al. 2009; Pridgeon et al. 2009), usually growing in cooler climates at high elevation. With the establishment of Swartz (1799), various generic delimitations and infrageneric systems have been proposed in this genus (Schlechter 1924; Hunt 1970; Seth and Cribb 1984; Puy and Cribb 1988; Liu et al. 2006; Chen et al. 2009). There are approximately 80 species all over the world, China is the diversity center of this genus, with about 50 species, 20 among which are endemic (Liu et al. 2006; Chen et al. 2009; Chen et al. 2019; Zhang et al. 2019).

Nowadays, about 15 complete chloroplast genome of *Cymbidium* have been reported, such as *C. nanulum* (Zhang et al. 2019), *C. eburneum* (Wang et al. 2019), and *C. erythraeum* (Huang et al. 2019). The data of complete chloroplast genome will serve as a foundation for species identification, germplasm diversity, genetic engineering of *Cymbidium*. In this study we reported the complete chloroplast genome sequence of *C. mastersii.*

Leaf samples of *C. mastersii* were obtained from the Orchid Conservation and Research Centre of Shenzhen, and specimens were deposited in the National Orchid Conservation Center herbarium (NOCC; specimen code Z.J.Liu 2924). Total genomic DNA was extracted from fresh material using the modified CTAB procedure of Doyle and Doyle (1987). Sequenced on Illumina Hiseq 2500 platform (Illumina, San Diego, CA). Genome sequences were screened out and assembled with MITObim v1.8 (Hahn et al. 2013), which resulted in a complete circular sequence of 155,362 bp in length. The cp genome was annotated with CpGAVAS (Liu et al. 2012).

The cp genome sequence of *C. mastersii* (GenBank accession MK848042) was 155,362 bp in length and presented a typical quadripartite structure including one large single-copy region (LSC, 84,465 bp), one small single-copy region (SSC, 20,647 bp), and two inverted repeat regions (IRs, 25,125 bp). The cp genome encoded 130 genes, of which 107 were unique genes (80 protein-coding genes, 23 tRNAs, and 4 rRNAs).

The complete cp genome sequences of 15 species from the genus *Cymbidium* were utilized to clarify the phylogenetic position of *C. mastersii*, and the *Pleione formosana* and *P. bulbocodioides* were treated as outgroups. The sequences were aligned by using MAFFT v7.307 (Katoh and Standley 2013), maximum-likelihood (ML) analysis was conducted using the RAxML software (Stamatakis 2014) with 1000 bootstrap replicates. The result showed that they were all clustered together (Figure 1), and *C. mastersii* was a sister to *C. erythraeum* and *C. nanulum*. This report will open up avenues for further research to understand the genomic information of the chloroplasts of the genus *Cymbidium* and further study on application in phylogeny, species identification and conservation genetics.

**Figure 1.**
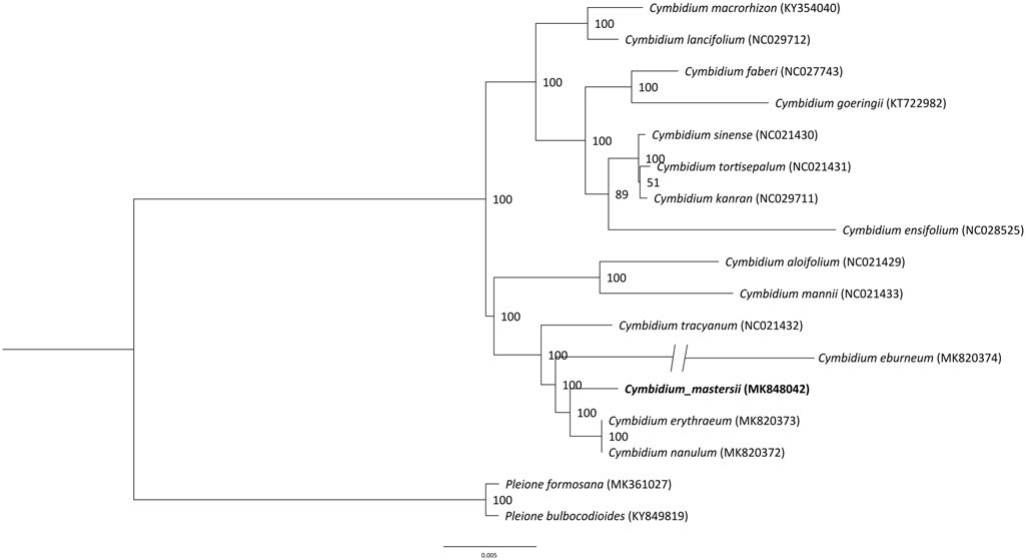
Phylogenetic position of *Cymbidium mastersii* inferred by maximum-likelihood (ML) of complete cp genome. The bootstrap values are shown next to the nodes.

## References

[CIT0001] Chen GZ, Zhang GQ, Huang J, Wang M, Rao WH, Chen LJ 2019 *Cymbidium shidianense* (Orchidaceae: Epidendroideae), a new species from China: evidence from morphology and molecular data. Phytotaxa. 399:100–108.

[CIT0002] Chen SC, Liu ZJ, Zhu GH, Lang KY, Ji ZH, Luo YB, Jin XH, Cribb PJ, Wood JJ, Gale SW, et al. 2009 Orchidaceae In: Wu ZY, Raven PH, Hong D, editors. Flora of China, vol. 25. St. Louis (MO): Science Press, Beijing & Missouri Botanical Garden Press; p. 260.

[CIT0003] Doyle JJ, Doyle JL 1987 A rapid DNA isolation procedure from small quantities of fresh leaf tissue. Phytochem Bull. 19:11–15.

[CIT0004] Hahn C, Bachmann L, Chevreux B 2013 Reconstructing mitochondrial genomes directly from genomic next-generation sequencing reads-a baiting and iterative mapping approach. Nucleic Acids Res. 41:e129. 23661685 10.1093/nar/gkt371 PMC3711436

[CIT0005] Huang J, Chen GZ, Li TZ, Huang ZC, Rao WH, Chen JB 2019 The complete chloroplast genome of *Cymbidium erythraeum* (Orchidaceae). Mitochondrial DNA Part B. 4:2517–2518. 10.1080/23802359.2019.1638322 PMC7687648 33365607

[CIT0006] Hunt PF 1970 Notes on Asiatic orchid 5. Kew Bull. 24:93–94.

[CIT0007] Katoh K, Standley DM 2013 MAFFT multiple sequence alignment software version 7: improvements in performance and usability. Mol Biol Evol. 30:772–780. 23329690 10.1093/molbev/mst010 PMC3603318

[CIT0008] Liu C, Shi LC, Zhu YJ, Chen HM, Zhang JH, Lin XH, Guan XJ 2012 CpGAVAS, an integrated web server for the annotation, visualization, analysis, and GenBank submission of completely sequenced chloroplast genome sequences. BMC Genomics. 13:715. 23256920 10.1186/1471-2164-13-715 PMC3543216

[CIT0009] Liu ZJ, Chen XQ, Ru ZZ, Chen LJ 2006 The genus Cymbidium in China. Beijing (China): Science Press.

[CIT0010] Pridgeon AM, Cribb PJ, Chase MW, Rasmussen FN 2009 Epidendroideae (Part two) Genera *Orchidacearum*, vol. 5. Oxford (UK): Oxford University Press.

[CIT0011] Puy DD, Cribb P 1988 The genus *Cymbidium*. Portland (OR): Timber Press.

[CIT0012] Schlechter R 1924 Die Gattungen *Cymbidium* Sw. und *Cyperorchis* Bl. Feddes Repert. 20:96–110.

[CIT0013] Seth CJ, Cribb PJ 1984 A reassessement of the sectional limits in the genus *Cymbidium* Swartz In: Arditti J., editor. Orchid biology, reviews and prospectives 3. London (UK): Cornell University Press; p. 283–322

[CIT0014] Stamatakis A 2014 RAxML version 8: a tool for phylogenetic analysis and post-analysis of large phylogenies. Bioinformatics. 30:1312–1313. 24451623 10.1093/bioinformatics/btu033 PMC3998144

[CIT0015] Swartz O 1799 Cymbidium grandiflorm. Nova Acta Societatis Scientiarum Upsaliensis. 6:73.

[CIT0016] Wang MJ, Chen JB, Chen GZ, Chen LJ, Wu XY, Huang J 2019 The complete chloroplast genome of *Cymbidium eburneum* (Orchidaceae). Mitochondrial DNA Part B. 4:2359–2360. 10.1080/23802359.2019.1629844 PMC7687489 33365543

[CIT0017] Zhang GQ, Chen GZ, Chen LJ, Lan SR 2019 *Cymbidium yunnanensis*: a new orchid species (Orchidaceae; Epidendroideae) from China based on morphological and molecular evidence. Phytotaxa. 387:149–157.

[CIT0018] Zhang YQ, Li TZ, Huang J, Chen GZ, Zhang GQ 2019 The complete chloroplast genome of an endangered species *Cymbidium nanulum* (Orchidaceae). Mitochondrial DNA Part B. 4:2497–2498. 10.1080/23802359.2019.1638327 PMC7687592 33365599

